# Supplementary material for: A Small De Novo CNV Deletion of the Paternal Copy of FOXF1, Leaving lncRNA FENDRR Intact, Provides Insight into Their Bidirectional Promoter Region
Source: Noncoding RNA. 2023 Oct 9;9(5):61. doi: 10.3390/ncrna9050061 (PMC10609350; doi:10.3390/ncrna9050061)

SUPPLEMENTARY MATERIALS

A small *de novo* CNV deletion of the paternal copy of *FOXF1,* leaving lncRNA *FENDRR* intact, provides insight into their bidirectional promoter region

Przemyslaw Szafranski and Paweł Stankiewicz

Clinical report

The proband (pt 219.3) was born as the first child, full term at 38+4, through vaginal delivery. He weighed 3,195 g and measured 50 cm in length. The pregnancy and delivery were uneventful. His Apgar scores were 9, 10, 10 at 1, 5, and 10 min, respectively. On day 2, his oxygen level declined rapidly and he received CPAP with oxygen. He responded well to the oxygen and did not need the mask the following day. The next morning, during breast feeding, he was turning slightly blue around his mouth. The oxygen CPAP did not help and he was stabilized with nitrogen oxide under sedation and on ventilation. Subsequent ultrasound and x-rays showed no abnormalities. However, an echocardiogram showed functionally dilated right ventricle likely due to persistent pulmonary hypertension and a TI of 60-65 mm Hg. Otherwise, there was no evidence of any structural heart anomaly. The aortic valve was tricuspid and slender, and no aortic insufficiency was noticed. When his oxygen levels started to deteriorate again, he had to be transferred to ECMO. Life support was terminated and he passed away at age of six days.

Table S1. Primers used to amplify fragments of the bi-directional *FOXF1* & *FENDRR* promoter region for the luciferase assay.

Promoter fragment name	Forward primer (5'-3')	Reverse primer (5'-3')	GRCh38/hg38 coordinates of the amplified region	Size of the amplified fragment (bp)
A	ATACTCGAGCGCGCTGTCTGCTCTCAGGAAC	ATAGCTAGCGCGGCCGCTTTGTCTCG	86,508,848-86,509,526	679
B	ATACTCGAGCGCGCTGTCTGCTCTCAGGAAC	ATAGCTAGCGGCACCACCTCCAGGTCTTACG	86,508,848-86,510,165	1,318
C	ATACTCGAGCGCGCTGTCTGCTCTCAGGAAC	ATAGCTAGCGTGCGGTGGCTGCTGCTTCTC	86,508,848-86,510,605	1,758
D	ATACTCGAGTTACCGGGTTTGCATGTGCATC	ATAGCTAGCGGCACCACCTCCAGGTCTTACG	86,509,333-86,510,165	833
E	ATAGCTAGCTTACCGGGTTTGCATGTGCATC	ATACTCGAGGTGCGGTGGCTGCTGCTTCTC	86,509,333-86,510,605	1,273
F	ATAGCTAGCCGCGCTGTCTGCTCTCAGGAAC	ATACTCGAGGTGCGGTGGCTGCTGCTTCTC	86,508,848-86,510,605	1,758

Figure S1. IGV view of the Illumina genome sequencing across the *FOXF1* deletion in the proband 219.3.

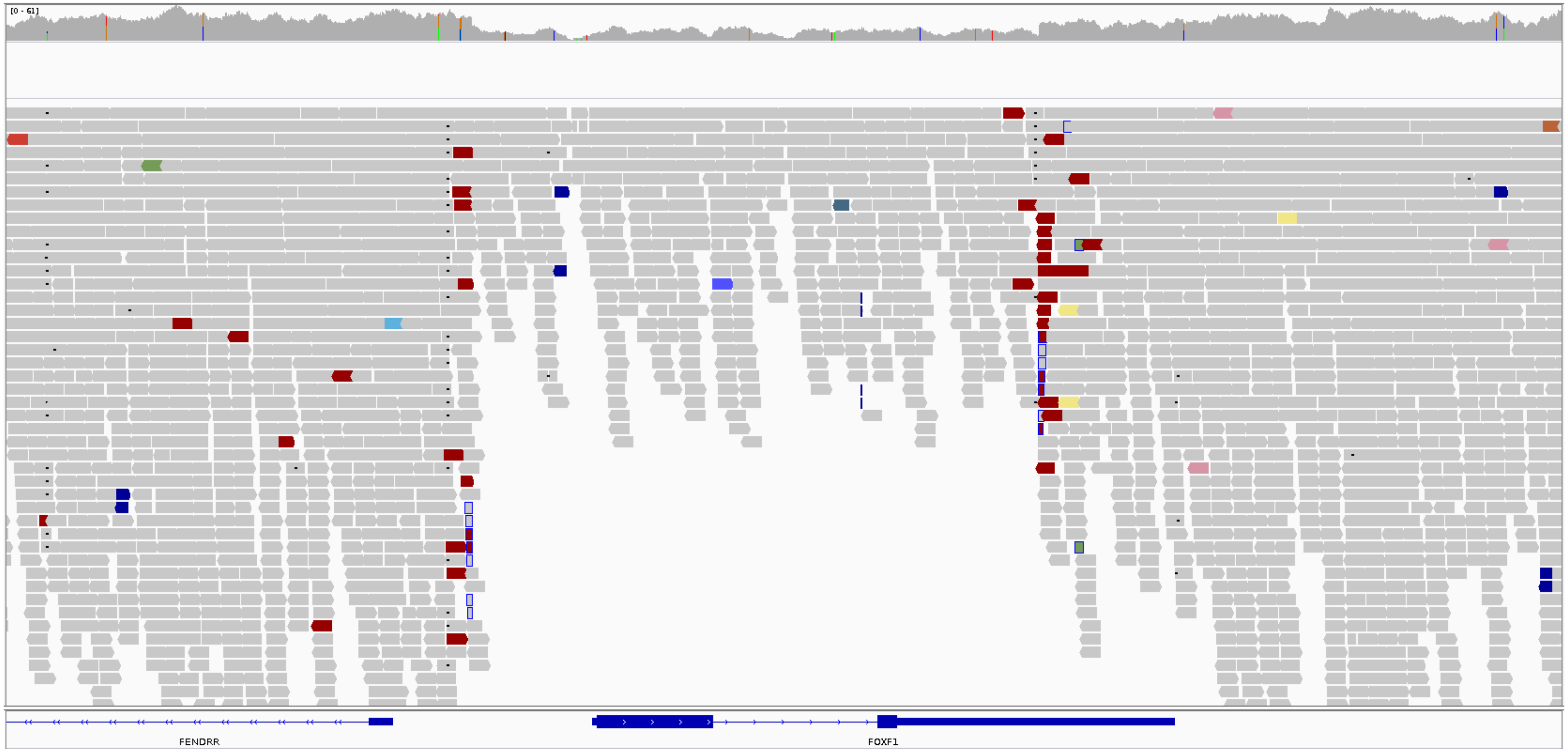

Figure S2. DNA sequence across the deletion junction. Microhomology (C) is on black background.

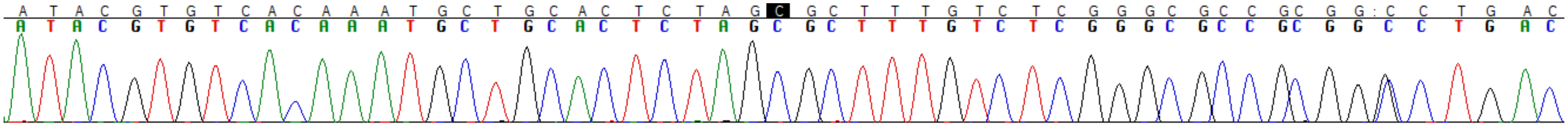

Figure S3. The deletion in the proband 219.3 occurred *de novo*. Two different primer pairs were used (left and right panels, respectively) to amplify the deletion junction: 5'-GAGTGACTGAGC ACGCCGCTTATT-3' / 5'-CCTCTATCTTTGGGAGTCTGGAAGGTAT-3' (left panel) and 5'-AGTGAGTGCGAACTTAAGCTCCTGTG-3' / 5'-CCTCTATCTTTGGGAGTCTGGAAGGT AT-3' (right panel).

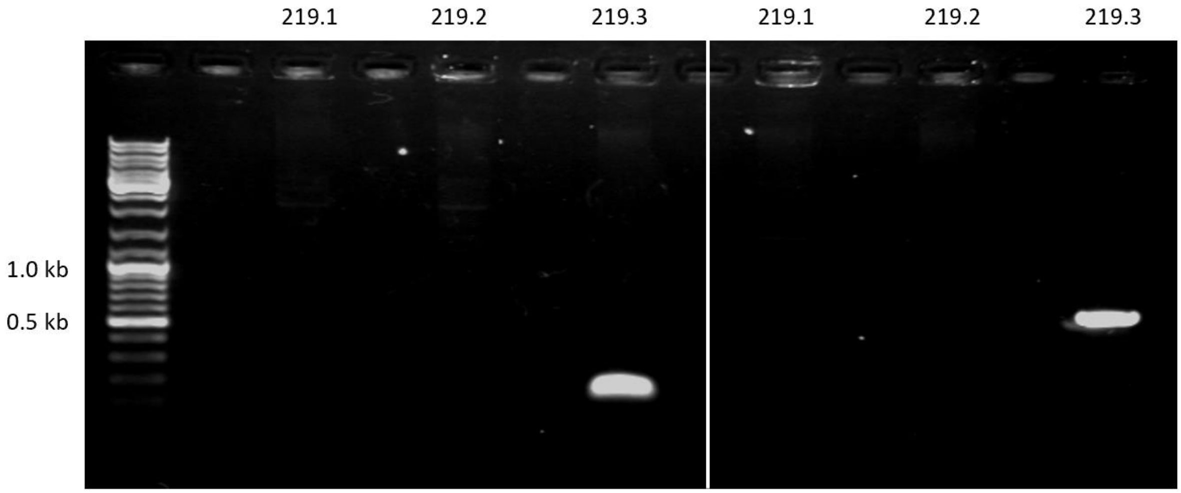

Figure S4. The deletion occurred on chr16 inherited from the father. Chromatograms represent Sanger sequencing in both directions of the informative SNV.

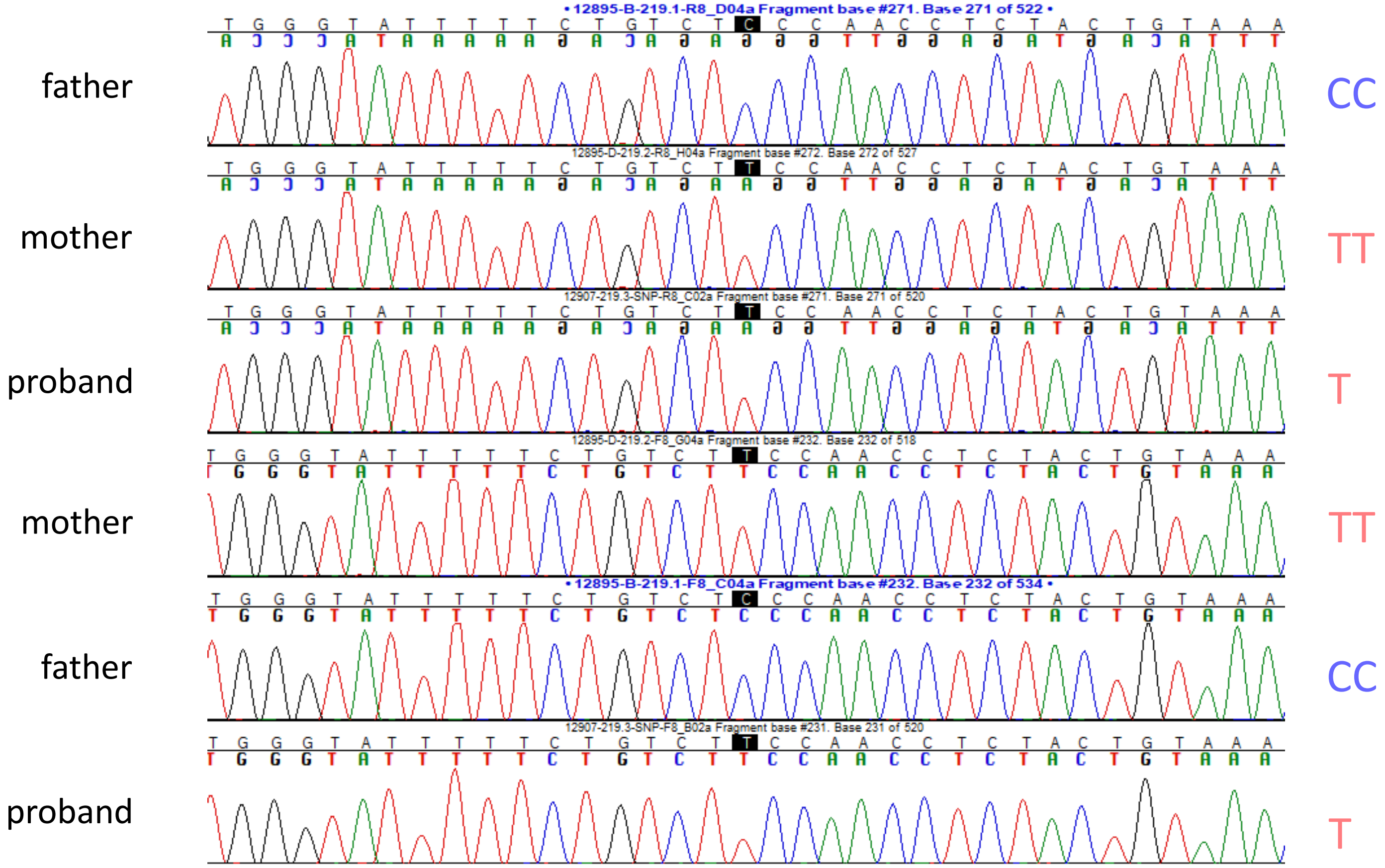

Supplement: Supplementary file 1 [file ncrna-09-00061-s001.zip › ncrna-2605979-supplementary.pdf]
